# Malnutrition and the disproportional burden on the poor: the case of Ghana

**DOI:** 10.1186/1475-9276-6-21

**Published:** 2007-11-28

**Authors:** Ellen Van de Poel, Ahmad Reza Hosseinpoor, Caroline Jehu-Appiah, Jeanette Vega, Niko Speybroeck

**Affiliations:** 1 Department of Applied Economics, Erasmus University Rotterdam, Burg. Oudlaan 50, 3000 DR Rotterdam, The Netherlands; 2 The Faculty of Economics and Commerce, The University of Melbourne, Victoria 3010, Australia; 3 Equity, Poverty and Social Determinants of Health, Evidence and Information for Policy, World Health Organization, Avenue Appia 20, CH - 1211 Geneva 27, Switzerland; 4 Policy Planning Monitoring and Evaluation Division, Ghana Health Service, Private Mail Bag, Ministries, Accra, Ghana; 5 Institute of Tropical Medicine, Nationalestraat 155, 2000 Antwerp, Belgium

## Abstract

**Background:**

Malnutrition is a major public health and development concern in the developing world and in poor communities within these regions. Understanding the nature and determinants of socioeconomic inequality in malnutrition is essential in contemplating the health of populations in developing countries and in targeting resources appropriately to raise the health of the poor and most vulnerable groups.

**Methods:**

This paper uses a concentration index to summarize inequality in children's height-for-age z-scores in Ghana across the entire socioeconomic distribution and decomposes this inequality into different contributing factors. Data is used from the Ghana 2003 Demographic and Health Survey.

**Results:**

The results show that malnutrition is related to poverty, maternal education, health care and family planning and regional characteristics. Socioeconomic inequality in malnutrition is mainly associated with poverty, health care use and regional disparities. Although average malnutrition is higher using the new growth standards recently released by the World Health Organization, socioeconomic inequality and the associated factors are robust to the change of reference population.

**Conclusion:**

Child malnutrition in Ghana is a multisectoral problem. The factors associated with average malnutrition rates are not necessarily the same as those associated with socioeconomic inequality in malnutrition.

## Background

In the developing world, an estimated 230 million (39%) children under the age of five are chronically malnourished and about 54% of deaths among children younger than 5 are associated with malnutrition [1]. Malnutrition is a major public health and development concern with important health and socioeconomic consequences. In Sub-Saharan Africa, the prevalence of malnutrition among the group of under-fives is estimated at 41% [1]. It is the only region in the world where the number of child deaths is increasing and in which food insecurity and absolute poverty are expected to increase [2-4]. Malnutrition in early childhood is associated with significant functional impairment in adult life, reduced work capacity and decreasing economic productivity [5-10]. Children who are malnourished not only tend to have increased morbidity and mortality but are also more prone to suffer from delayed mental development, poor school performance and reduced intellectual achievement [6-8].

Chronic malnutrition is usually measured in terms of growth retardation. It is widely accepted that children across the world have much the same growth potential, at least to seven years of age. Environmental factors, diseases, inadequate diet, and the handicaps of poverty appear to be far more important than genetic predisposition in producing deviations from the reference. These conditions, in turn, are closely linked to overall standards of living and the ability of populations to meet their basic needs. Therefore, the assessment of growth not only serves as one of the best global indicators of children's nutritional status, but also provides an indirect measurement of the quality of life of an entire population [11-13].

Large scale development programs such as the Millennium Development Goals (MDGs) have also emphasized the importance of the under-fives' nutritional status as indicators for evaluating progress [14]. When aiming at reducing childhood malnutrition, it is important not only to consider averages, which can obscure large inequalities across socioeconomic groups. Failure to tackle these inequalities may act as a brake on making progress towards achieving the MDGs and is a cause of social injustice [15,16].

## Ghana

Against this background, Ghana provides an interesting case study. The country experienced remarkable gains in health from the immediate post independence era. Life expectancy improved over the years and the prevention of a range of communicable diseases improved child survival and development. However in the last decade despite increasing investments in health, Ghana has not achieved target health outcomes. There has been no significant change in Ghana's under-five and infant mortality rates between 1993 and 2003. In the last couple of years, under-five mortality was actually slightly increasing. Life expectancy has also fallen from 57 years in 2000 to 56 years in 2005 [17]. Ghana's Human Development Index (HDI), a measure combing life expectancy, literacy, education and standard of living, has been worsening too; after improving from 0.444 in 1975 to 0.563 in 2001, the HDI dropped to 0.520 in 2005 [15]. Since 1988, there has been no definite trend in malnutrition (in terms of height-for-age). Apparent gains between 1988 and 1998 were reversed in 2003 [18]. Although the 2003 Ghana Demographic Health Survey (DHS) final report [17] recommends caution when using data from the various DHS to assess the trend in the nutritional status, it is noted that there was a trend over the past five years of increased stunting compared to a decrease of wasting and underweight. Further, there has been a trend of continued high values of stunting in the North compared to the South [17,19].

Malnutrition in Ghana has been most prevalent under the form of Protein Energy Malnutrition (PEM), which causes growth retardation and underweight. About 54% of all deaths beyond early infancy were associated with PEM, making this the single greatest cause of child mortality in Ghana [20].

A paradigm shift in Ghanaian health policy has been taking place in 2006. The theme for the new health policy in Ghana was 'Creating Wealth through Health". One of the fundamental hypotheses of this policy was that improving health and nutritional status of the population would lead to improved productivity, economic development and wealth creation [21]. Since this policy adopted an approach that addressed the broader determinants of health, it has thus generated interest in socio-economic inequalities in health and malnutrition. It was further recognised that not paying attention to malnutrition inequalities during the early years of life is likely to perpetuate inequality and ill health in future generations and thus defeat the aims of the new health policy.

From the existing evidence it is clear that childhood malnutrition is associated with a number of socioeconomic and environmental characteristics such as poverty, parents' education/occupation, sanitation, rural/urban residence and access to health care services. Also demographic factors such as the child's age and sex, birth interval and mother's age at birth have been linked with malnutrition [4,5,22-26]. Previous studies have also drawn attention to the disproportional burden of malnutrition among children from poor households [27-31]. However, much less is known on which factors lie behind this disproportional burden. It is important to note that the most important determinants of malnutrition are not necessarily also the most important determinants of socioeconomic inequality in malnutrition. [31] shows that the poorest-to-richest odds-ratio of stunting is almost halved by controlling for household and child characteristics using Ghanaian data. However, it is not clear how much each of these characteristics is contributing to this reduction. Understanding the nature and determinants of socioeconomic inequality in malnutrition is essential in contemplating the health of populations in developing countries and in targeting resources appropriately to raise the health of the poor and most vulnerable groups. This paper employs a concentration index to summarize inequality across the entire socioeconomic distribution rather than simply comparing extremes as in ratio measures. The concentration index is decomposed using the framework suggested by [32], allowing to identify the factors that are associated with socioeconomic inequality in malnutrition. This decomposition takes into account that both the association of a determinant with malnutrition as well as its distribution across socioeconomic groups play a role in the extent to which it is contributing to socioeconomic inequality in malnutrition. The usefulness of this approach has already been demonstrated on European data, but has known limited applications on developing countries.

Further, this paper contributes to the literature by delivering evidence on the determinants of malnutrition and socioeconomic inequality in Ghana using the new child growth standards population that has recently been released by the World Health Organization (WHO) [33]. This reference population includes children from Brazil, Ghana, India, Norway, Oman and the US. The new standards adopt a fundamentally prescriptive approach designed to describe how all children should grow rather than merely describing how children grew in a single reference population at a specified time [34]. For example, the new reference population includes only children from study sites where at least 20% of women are willing to follow breastfeeding recommendations. To our knowledge this is the first study presenting estimates of malnutrition in Ghana based upon these new standards. To check sensitivity of the results to this change in reference group, the analysis is also done using the US National Center for Health Statistics (NCHS) reference population [35].

The results are useful from a policy perspective as they can be used in setting policies to reduce malnutrition and the excessive burden on the poor. The results of this study are particularly relevant for Ghanaian policy makers, but can also be generalized to other settings in the sense that they show that malnutrition is associated with a broad range of factors and that the factors related to average malnutrition are not necessarily the same as those related to socioeconomic inequality in malnutrition.

## Methods

### Measuring malnutrition

Nutritional status was measured by height-for-age z-scores. An overview of other nutritional indices and why height-for-age is the most suited for this kind of analysis is provided in [36]. A height-for-age z-score is the difference between the height of a child and the median height of a child of the same age and sex in a well-nourished reference population divided by the standard deviation in the reference population. The new WHO child growth population is used as reference population [33]. To construct height-for-age z-scores based upon these standards, we used the software available on the WHO website [37]. To check sensitivity of the results to this change in reference group, the analysis is also done by using the US National Center for Health Statistics (NCHS) reference population [35].

Generally, children whose height-for-age z-score is below minus two standard deviations of the median of the reference population are considered chronically malnourished or stunted. In the regression models, the negative of the z-score is used as dependent variable (*y*). This facilitates interpretation since it has a positive mean and is increasing in malnutrition [32]. For the purpose of our analysis, using the z-score instead of a binary or ordinal variable indicating whether the child is (moderately/severely) stunted is preferred as it facilitates the interpretation of coefficients and the decomposition of socioeconomic inequality. However, binary indicators of stunting are also used in the descriptive analysis and to position Ghana within a set of other Sub-Saharan African countries.

### The concentration index as a measure of socioeconomic inequality

Assume *y*_*i *_is the negative of the height-for-age z-score of child *i*. The concentration index (C) of *y *results from a concentration curve, which plots the cumulative proportion of children, ranked by socioeconomic status, against the cumulative proportion of *y*. The concentration curve lies above the diagonal if *y *is larger among the poorer children and vice versa. The further the curve lies from the diagonal, the higher the socioeconomic inequality in nutritional status. A concentration index is a measure of this inequality and is defined as twice the area between the concentration curve and the diagonal. If children with low socioeconomic status suffer more malnutrition than their better off peers the concentration index will be negative [38]. It should be noted that the concentration index is not bounded within the range of [-1,1] if the health variable of interest takes negative, as well as positive values. Since children with a negative *y *are better off than children in the reference population, they cannot be considered malnourished. Therefore their z-score is changed into zero, such that the z-scores are restricted to positive values with zero indicating no malnutrition and higher z-scores indicating more severe malnutrition.

Further, the bounds of the concentration index depend upon the mean of the indicator when applied to binary indicators, such as stunting [39]. This would impede cross-country comparisons due to substantial differences in means across countries. To avoid this problem, we used an alternative but related concentration index that was recently introduced by [40] and does not suffer from mean dependence, when comparing Ghana with other Sub-Saharan African countries.

### Decomposition of socioeconomic inequality

More formally, a concentration index of *y *can be written as [38]:

$$C=\frac{2\sum_{i=1}^{n}y_{i}R_{i}}{\sum_{i=1}^{n}y_{i}}-1$$

where *y*_*i *_refers to the height-for-age of the *i*-th individual and *R*_*i *_is its respective fractional rank in the socioeconomic distribution. As will be discussed further in the following section, the present paper uses a continuous wealth variable, developed by principal component analysis, as a measure of socioeconomic status [see e.g. [41]].

If *y*_*i *_is linearly modelled

$$y_{i}=\alpha +\sum_{k=1}^{K}\beta_{k}x_{ik}+\varepsilon_{i},$$

[32] showed that the concentration index of height-for-age can be decomposed into inequality in the determinants of height-for-age as follows:

$$C=\sum_{k=1}^{K}\left(\frac{\beta_{k}{\bar{x}}_{k}}{\mu }\right)C_{k}+\frac{GC_{\varepsilon }}{\mu }$$

where μ is the mean of *y*, ${\bar{x}}_{k}$ is the mean of *x*_*k*_, *C*_*k *_is the concentration index of *x*_*k *_(with respect to socioeconomic status) and *GCε *is the generalized concentration index of the residuals. The latter term reflects the socioeconomic inequality in height-for-age that is left unexplained by the model and is calculated as $GC_{\varepsilon }=\frac{2}{n}\sum_{i=1}^{n}\varepsilon_{i}R_{i}$.

As the DHS data have a hierarchical structure, with children nested in households and households nested within communities, we have also considered using multilevel models to estimate the associations of variables with childhood malnutrition (see e.g. [42]). Allowing for random effects on the household and/or community level yielded coefficients that were similar to the ones from OLS regression corrected for clustering. Because of this similarity and because the use of multilevel models would complicate the decomposition of socioeconomic inequality in malnutrition, the remainder is based on results from linear regression corrected for clustering on the community level.

All estimation takes account of sample weights (provided with the DHS data). Statistical inference on the decomposition results is obtained through bootstrapping with 3000 replications. The bootstrap procedure takes into account the dependence of observations within clusters.

### Data

Data is used from the 2003 Ghana Demographic Health Survey (DHS) and are restricted to children under the age of 5. Anthropometric measures are missing for 12.3% of children in this age group. The final sample contains information on 3061 children. We did examine possible selection problems due to the high proportion of missing observations. A logit model explaining the selection in the sample and a Heckman sample selection model (using different exclusion restrictions) were used to check for this [43]. Both tests did not reveal large sample selection problems, and coefficients in the Heckman model were very similar to those in the model presented here.

The nutritional status of a child is specified to be a linear function of child-level characteristics such as age, sex, duration of breastfeeding, size at birth; maternal characteristics such as education, mother's age at birth, birth interval, marital status, use of health services, occupation and finally household-level characteristics such as wealth, type of toilet facility, access to safe water, number of under-five children in the household, region and urbanization. We preferred not to include information on the type of toilet and water source into the wealth indicator, as these variables can be expected to have a direct relation with children's growth apart from being correlated with household socioeconomic status [44].

The explanatory variables are described in the last column of Table 1. All have well documented relevance in the literature [5,22-26,31,32,45,46].

**Table 1:** Mean, standard deviation and description of all variables

Variable	Mean	SD	Description
Stunting (WHO)	0.36	0.48	Height-for-age z-score<-2SD of WHO population (1-0)
Z-score (WHO)	1.58	1.27	Height for age z-score (based upon WHO)
Stunting (NCHS)	0.29	0.45	Height-for-age z-score<-2SD of NCHS population (1-0)
Z-score (NCHS)	1.41	1.17	Height for age z-score (based upon NCHS)

Breastfeeding	16.98	8.34	Duration of breastfeeding (in months)

Age of child			
**≤ 6 months**	0.12	0.33	Age of child split into 3 categories: **≤ 6 months**; 6–12 months, >12 months
6–12 months	0.12	0.32	
> 12 months	0.76	0.43	

Size of child			
Size large	0.41	0.49	Size of child at birth in 4 categories: very large, large, **normal**, small, very small
**Size normal**	0.41	0.49	
Size small	0.12	0.32	
Size very small	0.06	0.24	

Sex of child	0.50	0.50	Sex of child: male(1), female (0)

Region			
Upper	0.09	0.29	region of residence: Upper (Upper East and Upper West), Middle (Ashanti and Brong Ahafo), South (Western, Central, Volta and Eastern), Accra, **Northern **[55]
Middle	0.30	0.46	
South	0.36	0.48	
Accra	0.11	0.31	
**Northern**	0.14	0.34	

Urban	0.33	0.47	Urban location (1), rural location (0)

Wealth			
**Poor**	0.39	0.49	Wealth groups (**poor) **based upon principal component analysis. The wealth indicator is estimated on household level and combines the following assets: electricity, radio, TV, fridge, bike, motor, car, phone and the type of the flooring material [60].
Middle	0.32	0.47	
Rich	0.29	0.45	

Toilet	0.70	0.46	Having a toilet (flush toilet, traditional pit toilet, ventilated improved pit latrine) (1-0)

Water	0.61	0.49	Whether the household has access to safe water available (1-0). The following sources of water supply were regarded as safe water: piped water (piped into dwelling, piped into yard, plot, or public tap); water from protected well

Twoplus	0.59	0.49	Whether there are more than two under-fives in the household (1-0)

Riskintb	0.10	0.30	Whether there were less than 24 months between the child's birth and the birth of the previous child (1-0)

Married	0.91	0.29	Whether the child's mother is married or living together (1-0)

Mother's education			
No or incomplete	0.56	0.50	Mother's education level split into 3 categories: no or incomplete primary, primary and incomplete secondary, **secondary and higher**
Primary	0.40	0.49	
**Secondary and higher**	0.04	0.20	

Health services index			
**Healthlow**	0.33	0.47	Use of health services (**low**, moderate, high) estimated by principal component analysis. The indicator combines skilled birth attendance, antenatal care and proportion of recommended vaccinations [45]. The age schedule from the Expanded Program on Immunization set by the WHO was used: BCG at birth, DPT and Polio at 2, 3 and 4 months and measles at 9 months.
Healthmod	0.32	0.46	
Healthhigh	0.31	0.46	

Mother's age at birth			
<20	0.11	0.31	Mother's age at birth in years split into 3 categories: <20, **20–39**, >39
**20–39**	0.81	0.39	
>39	0.08	0.27	

Mother's occupation			
Prof, tech, man, cler, sales, service	0.32	0.47	Professional, technical, managerial, clerical, sales, services; agriculture; manual; **not working**
Agriculture	0.44	0.50	
Manual	0.14	0.35	
**Not working**	0.10	0.30	

Observations	3061		

Reference categories for categorical variables used in the regression model are in bold.

No information on mother's nutritional status was included in the set of explanatory variables. Since about 10% of women in the dataset were pregnant at the time of interview, their BMI did not provide an accurate measure of their nutritional status. Furthermore, BMI reflects current nutritional status and may not be relevant for children born 5 years prior to the interview. Inclusion of mother's height-for-age had no significant effect on results.

## Results

### Summary statistics

In the 2003 DHS data for Ghana, 36% of children under the age of 5 are stunted. Stunting is defined as height-for-age being below minus 2 SD from the median of the reference population. The concentration index for stunting in children under the age of 5 was -0.12 (SD = 0.016). This negative value implies that poor children had a higher probability of being stunted than their better off peers. Using the older NCHS reference study showed a lower prevalence of stunting (29%) and slightly higher socioeconomic inequality (C = -0.15, SD = 0.019).

**Figure 1:**
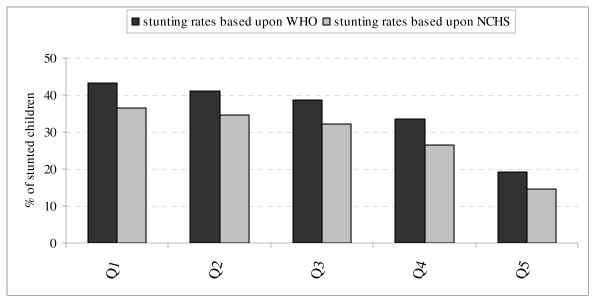
Distribution of stunting across wealth quintiles.

Figure 1 illustrates the strong socioeconomic inequality in childhood stunting. The stunting rate among the poorest 60 percent was more than twice the rate of children in the richest 20 percent.

**Figure 2:**
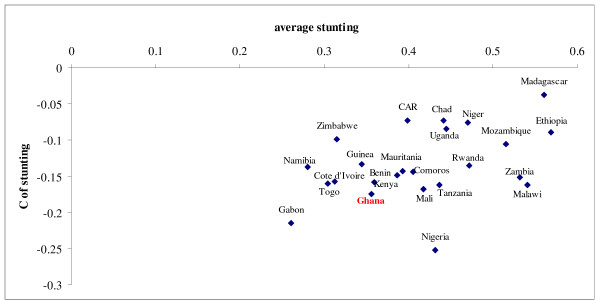
Average stunting versus socioeconomic inequality in stunting in under-five children in Sub-Saharan Africa. Data from recent Demographic Health surveys. Stunting is measured using the WHO child growth standards. Concentration index as suggested by [40] is used since it is invariant to the mean of the binary variable.

Figure 2 shows a comparative picture of stunting and socioeconomic inequality in stunting across the Sub-Saharan African region. Stunting and socioeconomic variables are calculated for each country on DHS data in exactly the same way as is described for the Ghana DHS. Summary statistics of all variables are shown in Table 1.

### Determinants of malnutrition

The regression coefficients and their significance are shown in the first column of Table 2. Note that the dependent variable is increasing in malnutrition, such that a negative coefficient should be interpreted as lowering malnutrition.

**Table 2:** Regression and decomposition results: coefficient, concentration index (C) and proportional contribution

Variables	Coefficient	C	Contribution (%)	Contribution (%)
Breastfeeding	**0.01**	-0.0042	0.54	0.54

Age of child				**-8.14**
6–12 months	**0.22**	0.0049	-0.10	
> 12 months	**0.86**	**0.0154**	**-8.04**	

Size of child				2.01
Size large	**-0.12**	0.0170	0.65	
Size small	**0.18**	-0.0500	0.82	
Size very small	**0.26**	-0.0401	0.54	

Sex of child	**0.23**	-0.0101	0.92	0.92

Region				**23.07**
Upper	**-0.59**	**-0.2123**	**-8.29**	
Middle	**-0.38**	**0.1169**	**10.34**	
South	**-0.52**	**-0.0425**	-6.68	
Accra	**-0.73**	**0.4390**	**27.70**	

Urban	-0.11	**0.3153**	8.95	8.95

Wealth				**30.85**
Middle	-0.04	**0.1055**	1.13	
Rich	**-0.18**	**0.7120**	**29.71**	

Toilet	-0.10	**0.1159**	6.71	6.71

Water	0.02	**0.0690**	-0.72	-0.72

Twoplus	**0.11**	**-0.0469**	**2.41**	**2.41**

Riskintb	**0.19**	0.0440	-0.66	-0.66

Married	-0.03	**0.0180**	0.35	0.35

Mother's education				5.51
No or incomplete	**0.33**	**-0.1578**	**22.99**	
Primary	**0.36**	**0.1549**	**-17.48**	

Health services index				**18.32**
Healthmod	-0.02	**-0.0525**	-0.20	
Healthhigh	**-0.32**	**0.2204**	**18.52**	

Mother's age at birth				1.29
<20	**0.13**	**-0.1133**	1.26	
>39	0.00	**-0.1035**	0.03	

Mother's occupation				2.90
Prof, tech, man, cler, sales, service	-0.13	**0.2194**	7.40	
Agriculture	-0.07	**-0.1884**	-4.90	
Manual	-0.07	0.0505	0.40	
Constant	**1.03**			

Error		**-0.0045**	**5.70**	**5.70**

Total			**100.00**	100.00

The dependent variable in the regression is the (negative) height-for-age z-score (based upon the WHO reference population). Number of observations = 3061, C of dependent variable = -0.079. Bold numbers indicate significance at the 10% level (based upon bootstrapped standard errors).

Malnutrition increased with the child's age in a non-linear way. Children who were very small at birth had a higher probability to be stunted than children with normal size. Male children were more prone to malnutrition than their female peers. Long duration of breastfeeding is associated with higher malnutrition.

**Figure 3:**
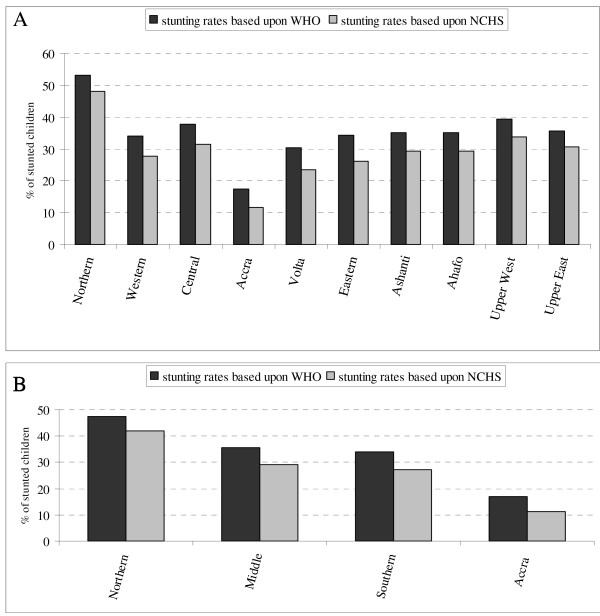
Inequality in stunting by regions (A) and grouped regions (B) (as in [55]).

With respect to maternal characteristics, the existence of a short birth interval was significantly increasing malnutrition. Children of women that accessed health services more frequently were less prone to being malnourished. Maternal occupation showed no clear effect. Maternal education and household wealth showed a significant association with childhood malnutrition. The presence of two or more under-five children in the household was negatively associated with the child's nutritional status. Sanitation variables however had no significant association on malnutrition. As compared to the Northern region all regions were associated with lower malnutrition, especially the Accra region. The high regional disparities in malnutrition are further illustrated in Figure 3. The four most deprived regions in Ghana (Northern, Central, Upper East and Western regions) exhibited the greatest burden of malnutrition.

### Decomposition of socioeconomic inequality in malnutrition

Table 2 also shows the concentration index and the relative contributions of each determinant to socioeconomic inequality in childhood malnutrition. For the ease of interpretation, the last column shows the grouped contribution from the categorical variables. A negative contribution to socioeconomic inequality implies that the respective variable is lowering socioeconomic inequality and vice versa. A variable can contribute to socioeconomic inequality in malnutrition both through its association with malnutrition and through its unequal distribution across wealth groups. The extent to which each of the explanatory variables is unequally distributed across wealth is reflected by its C value. A negative C means that the determinant is more prevalent among poorer households.

Wealth accounted for the major part (31%) of socioeconomic inequality. This part of socioeconomic inequality reflects the direct contribution of wealth. The remainder is the wealth-related inequality in malnutrition through other factors. Important contributors were regional variables (23%) and the use of health care services (18%). The age of the child was contributing negatively to socioeconomic inequality (-8%). This means that the combined effect of its coefficient and its distribution by wealth was lowering socioeconomic inequality in malnutrition. Older children were more likely to be stunted and were more prevalent in higher wealth quintiles. The latter is reflected by the positive and significant C of the variable *age*>*12 months *The contribution of the error term only amounted to about 6%, meaning that the decomposition model functioned well in explaining socioeconomic inequality in malnutrition.

Using the older NCHS reference population gave very similar regression and decomposition results are therefore not discussed (results are available upon request.).

## Discussion

Relative to other Sub-Saharan countries, Ghana appeared to have a rather low level of average stunting, combined with relatively high socioeconomic inequality in stunting. The use of the new WHO child growth standards yielded a higher average stunting rate as compared to the older NCHS reference group. [47] found the same for Bangladesh, Dominican Republic and a pooled sample of North American and European children. However, the variables associated with malnutrition and socioeconomic inequality were very robust to the change of the reference population.

### Determinants of malnutrition

Malnutrition in Ghanaian children rises with the age of the child, which is confirmed by other studies [5,25,32]. The higher prevalence of malnutrition among boys as compared to girls, and the negative association of long breastfeeding have also been established in the literature [5,22,32,45]. Long duration of breastfeeding may be associated with higher malnutrition because it reflects lack of resources to provide children with adequate nutrition [31]. It is also possible that children who are breastfed for a long time are more reluctant to eat other foods, as was found by [22] in their study on a cohort of Ghanaian children.

Short birth intervals and the presence of two or more under-five children in the household, affected childhood growth negatively by placing a heavy burden on the mother's reproductive and nutritional resources, and by increasing competition for the scarce resources within the household [22]. Children of younger mothers could be more prone to malnutrition because of physiological immaturity and social and psychological stress that come with child bearing at young age [48].

Maternal education was significantly lowering childhood malnutrition. This may reflect education generating the necessary income to purchase food. However, although education is often suggested to be a measure of social status, the coefficient stayed significant after controlling for household wealth and living conditions. A high level of maternal education could also lower childhood malnutrition through other pathways such as increased awareness of healthy behaviour, sanitation practices and a more equitable sharing of household resources in favour of the children [4,5,49].

Sanitation in terms of having a toilet and access to safe water did not significantly affect malnutrition. [26] also reported this result, but they did find a significant association between sanitation and wasting (which reflects current nutritional status). This might suggest that good sanitation can avoid episodes of diarrhoea and hereby affect current nutritional status, while it may not be sufficient for long term child growth.

The higher levels of malnutrition of the population living in the northern regions of Ghana have already been observed more than a decade ago [23]. This regional pattern reflects ecological constraints, worse general living conditions and access to public facilities in the Northern regions. In addition, the persistence of this regional inequality can point to an intergenerational effect of malnutrition. Since women who were malnourished as children are more likely to give birth to low-birth-weight children, past prevalence of child malnutrition is likely to have an effect on current prevalence.

### Decomposition of socioeconomic inequality in malnutrition

The high socioeconomic inequality in childhood malnutrition is -apart from wealth itself-mainly associated with regional characteristics and use of health care services. Wealth was responsible for about one third of the socioeconomic inequality in malnutrition. This means that poorer children were more likely to be malnourished, mainly because of their poverty. The regional contribution results from the fact that poorer children are more likely to live in regions with disadvantageous characteristics. Given the strong regional associations with malnutrition, after controlling for a broad range of socioeconomic and demographic covariates, there must be other important regional aspects. The regional inequality in Ghana originates from both geographical and historical reasons. Much of the North is characterized by lower rainfall, savannah vegetation, periods of severe drought and remote and inaccessible location. Further, the colonial dispensation ensured that northern Ghana was a labor reserve for the southern mines and forest economy and the post-colonial failed to break the established pattern [19].

Health services use was also responsible for a substantial proportion of socioeconomic inequality in malnutrition. This derives from the combined effect of the positive associations between health services use and childhood growth and the unequal use across socioeconomic groups. The reason for the lower health care use amongst the poor may be due to several barriers including the cost of care, cost of transportation and lower awareness on health promoting behavior [50]. User fees were introduced in Ghana in 1985 as a cost-sharing mechanism at all public health facilities. To ensure access to health care services for the poor and vulnerable the government introduced fee exemptions. Then again in 2003, a new policy for exempting deliveries from user fees in the four most deprived regions of the country, namely Central, Northern, Upper East and Upper West regions were introduced. To further bridge the inequality a key recommendation of the Ghana Poverty Reduction Strategy [51] was to allocate 40% of the non-wage recurrent budget to the deprived regions. However, experience to date indicates that Ghana has not been able to implement an efficient exemption mechanism or commit to the 40% budgetary allocation to achieve the principal purpose. In addition to these financial hurdles, poorer people are often also located further from health centers. The ratios of population to nurses and doctors are the highest the poorest regions of Ghana. For example the ratio of population to doctors in the northern region is 1:81338 compared to the national average of 1:17733. Trends show that since 1995 the Northern region has had the lowest average number of outpatient visits per capita in the country [52]. Also partly related to the use of health services is the contribution of the number of under-fives in the household. Poor women are more likely to have more children and these, in turn, are therefore more likely to be malnourished. The higher parity among poorer women may be related to difficult access to or knowledge on family planning services. The much lower use and knowledge of modern contraception among poor women is documented in the Ghana DHS 2003 final report [17].

The negative contribution of age comes from the combined facts that older children are more likely to be malnourished and at the same time more prevalent in the richer wealth quintiles. The latter could be related to higher child and infant mortality rates amongst poorer households that cause the proportion of older children to be lower among poor households as compared to richer households.

Combining the results from the analysis on the determinants of malnutrition and socioeconomic inequality demonstrates that variables that are associated with average malnutrition are not necessarily also related to socioeconomic inequality. Although bio-demographic variables such as a risky birth interval, size at birth, duration of breastfeeding and the sex of the child are quite strongly associated with a child's nutritional status, they do not contribute to socioeconomic inequality in malnutrition. This is because of their relatively equal distribution across socioeconomic groups. Other variables such as urban/rural location, having a toilet, access to clean water and maternal occupation are very unequally distributed across socioeconomic groups, but still do not contribute to socioeconomic inequality in malnutrition because they are not significantly associated with malnutrition. A third group of variables such as regions, health care use and wealth are both very strongly related to average and socioeconomic inequality in malnutrition.

### Considerations and limitations

There exist some limitations of this study. First, DHS only collects information on the recent food consumption of the youngest child under three years of age living with the mother. Restricting the sample to these children would substantially reduce the number of observations. However, the analysis was also conducted on this sub sample, using food consumption as one of the determinants of malnutrition (indices were created similar to [25,45]). Since the regression and decomposition results did not differ much, these are not presented in this paper (but are available from the authors upon request). Second, one has to bear in mind that, although commonly used, the construction of an asset index to capture socioeconomic status has its shortcomings and e.g. is sensitive to the assets included [44]. However, in the absence of reliable information on income or expenditure, the use of such an asset index is generally a good alternative to distinguish socioeconomic layers within a population [53]. Finally, it is important to note that this paper is showing the factors that are associated with malnutrition and socioeconomic inequality in malnutrition and the magnitude of these associations. These results are subject to the usual caveats regarding the causal interpretation of cross-sectional results. Focusing on child health avoids much of the direct feedback of income and health that is usually present in microeconomic studies. To gain some insight into the severity of endogeneity problems we also did the analysis excluding possible endogenous variables such as birth interval, breastfeeding, the number of children in the household and use of health care services. Again, wealth and regional characteristics were contributing most to socioeconomic inequality, followed by maternal education. To avoid endogeneity of health care use, it would be better to use data on proximity/availability of care. However, no such data were available in the 2003 Ghana DHS. Another option would be to predict health care use, but we were not able to find strong predictors for health care.

## Conclusion and policy implications

The regression results show that malnutrition is associated with a broad range of factors. However in Ghana it often falls through the cracks since it has no institutional home. Tackling malnutrition therefore calls for a shared vision and should be viewed and addressed in a broader context [54]. Therefore special attention needs to be given to policies aimed at reducing malnutrition based on the magnitude and nature of determinants of malnutrition, such as poverty, education, health care and family planning services and regional characteristics. Currently in Ghana, various interventions are being implemented to reduce both PEM and micro nutrient deficiencies. These include the Infant and Young Child Feeding Strategy (IYCF) and Community Based Nutrition and Food Security project among others. Notwithstanding the positive effects of these programs, they address only the symptoms of malnutrition and therefore are most likely not sufficient to have a sustained impact in the long term as they do not deal with a lot of the root causes of malnutrition.

The results also suggest that factors strongly associated with average malnutrition are not necessarily also contributing to socioeconomic inequality in malnutrition. The distinction between these groups of variables can be quite important, as it suggests that policies trying to reduce average malnutrition rates can be different from those aiming at lowering socioeconomic inequality in malnutrition. If equity goals are to be achieved, health policies in Ghana should further be directed at strategies/interventions to reduce poverty and to improve the use of health care and family planning services among the poorer population groups. Furthermore, regional disparities should further be tackled to narrow the gap in malnutrition between the poor and the rich. A starting point could be for policy makers to include under-five malnutrition differentials to set criteria to guide resource allocation to regions. Moreover, the strong regional contributions to socioeconomic inequality, even after controlling for other factors such as household wealth and education, bring forward the issue of geographical targeting. Further targeting public programs towards the central and northern regions would substantially reduce socioeconomic inequality in malnutrition and is administratively easier than targeting the poor. The latter argument is relevant for Ghana, where pro-poor policies (redistribution schemes and exemption policies) are not having the aimed effect because of problems in identifying the poor [55,56]. Geographic targeting reduces leakage of program benefits to the non-needy compared to untargeted programs, although under coverage of the truly needy can increase. "Fine-tuning" the targeting by basing it on smaller geographic units increases efficiency, but in some circumstances may be costly and politically unacceptable [57].

With respect to Ghana, regional averages should be interpreted with caution as there is large heterogeneity between districts in each region and indeed among socio-economic groups within districts. In this case, polices aimed at reducing child malnutrition based on regional averages may lead to under coverage of those in need. [58] exposes some important limitations of geographic targeting if used to place poverty-alleviation or nutrition interventions within cities. Using data from Abidjan (Cote d'Ivoire) and Accra (Ghana), they found significant clustering in housing conditions; however they did not find any sign of geographic clustering of nutritional status in either city. This implies that geographic targeting of nutrition interventions in these and similar cities has important limitations. Geographic targeting would probably lead to a significant under coverage of the truly needy and, unless accompanied by additional targeting mechanisms, would also result in significant leakage to non-needy populations. Nonetheless, there is a need for additional research to further decompose regional malnutrition inequalities to generate valuable information for policy making decisions. The Ghana Growth and Poverty Reduction Strategy for 2006 – 2009 [59] states that one of the strategies to be implemented is developing and implementing high impact yielding strategies for malnutrition. This would mean targeting areas at the greatest risks of malnutrition, replicate best practices and expand coverage. This then should result in decreasing malnutrition rates among children particularly in rural areas and northern Ghana.

## Authors' contributions

EVDP was responsible for the study design, analysis, interpretation of the data and writing of the paper. ARH and NS contributed to formulating the study design, interpreting the data and revising the manuscript. CJA contributed to the writing of the paper and revising the manuscript. JV provided guidance to the work and commented on the manuscript. All authors approved the final version of the paper.
